# Alendronate-induced disruption of actin cytoskeleton and inhibition of migration/invasion are associated with cofilin downregulation in PC-3 prostate cancer cells

**DOI:** 10.18632/oncotarget.25961

**Published:** 2018-08-24

**Authors:** Sanna S. Virtanen, Tamiko Ishizu, Jouko A. Sandholm, Eliisa Löyttyniemi, H. Kalervo Väänänen, Johanna M. Tuomela, Pirkko L. Härkönen

**Affiliations:** ^1^ University of Turku, Institute of Biomedicine, FI-20520 Turku, Finland; ^2^ Turku University of Applied Sciences, Health and Well-being, FI-20520 Turku, Finland; ^3^ Cell Imaging Core, Turku Centre for Biotechnology, University of Turku and Åbo Akademi University, FI-20521 Turku, Finland; ^4^ University of Turku, Department of Biostatistics, FI-20520 Turku, Finland

**Keywords:** bisphosphonate, prostate cancer, invasion, actin cytoskeleton, cofilin

## Abstract

Bisphosphonates are used for prevention of osteoporosis and metastatic bone diseases. Anti-invasive effects on various cancer cells have also been reported, but the mechanisms involved are not well-understood. We investigated the effects of the nitrogen-containing bisphosphonate alendronate (ALN) on the regulation of actin cytoskeleton in PC-3 cells. We analyzed the ALN effect on the organization and the dynamics of actin, and on the cytoskeleton-related regulatory proteins cofilin, p21-associated kinase 2 (PAK2), paxillin and focal adhesion kinase. Immunostainings of cofilin in ALN-treated PC-3 cells and xenografts were performed, and the role of cofilin in ALN-regulated F-actin organization and migration/invasion in PC-3 cells was analyzed using cofilin knockdown and transfection. We demonstrate that disrupted F-actin organization and decreased cell motility in ALN-treated PC-3 cells were associated with decreased levels of total and phosphorylated cofilin. PAK2 levels were also lowered but adhesion-related proteins were not altered. The knockdown of cofilin similarly impaired F-actin organization and decreased invasion of PC-3 cells, whereas in the cells transfected with a cofilin expressing vector, ALN treatment did not decrease cellular cofilin levels and migration as in mock transfected cells. ALN also reduced immunohistochemical staining of cofilin in PC-3 xenografts. Our results suggest that reduction of cofilin has an important role in ALN-induced disruption of the actin cytoskeleton and inhibition of the PC-3 cell motility and invasion. These data also support the idea that the nitrogen-containing bisphosphonates could be efficacious in inhibition of prostate cancer invasion and metastasis, if delivered in a pharmacological formulation accessible to the tumors.

## INTRODUCTION

Metastatic bone lesions are commonly associated with breast and prostate cancers, affecting approximately 65–80% of patients with an advanced disease [1]. Bisphosphonates (BPs) are widely used therapeutic agents for a variety of metabolic bone diseases, such as bone metastasis, due to their ability to inhibit bone resorption [2]. The first generationpyrophosphate-resembling BPs (e.g. clodronate and etidronate) are metabolized to toxic ATP analogues in osteoclasts [3–5]. Nitrogen-containing BPs (N-BPs; e.g. alendronate (ALN) and zoledronate) are more effective inhibitors of bone resorption, and their effects are largely mediated via inhibition of the mevalonate pathway and isoprenylation of important small GTPases such as the Ras, Rac and Rho family proteins [6]. Both farnesyl diphosphate synthase [7, 8] and geranylgeranyl diphosphate synthase [9] have been identified as N-BP targets in osteoclasts.

N-BPs also have anti-migratory and anti-adhesive effects on cancer cell lines [10, 11]. These changes were reported to be associated with inhibition of Rho GTPases [12]. We and others have previously shown that the N-BP ALN induces changes in F-actin organization and decreases cell attachment and migration in prostate cancer cells [6, 13]. Mönkkönen and coworkers have reported the accumulation of an endogenous ATP analogue (ApppI), and the induction of apoptosis by N-BPs in osteoclasts and in various breast and prostate cancer cell lines [14, 15]. N-BPs have also been shown to have anti-proliferative, anti-angiogenic and pro-apoptotic effects on prostate cancer cell lines [16–19]. *In vivo* N-BPs also have immunomodulatory [20–22], apoptotic, antimetastatic and tumor growth inhibiting effects [17, 23, 24].

Regulation of actin cytoskeleton is critical for cell motility [25, 26]. The reorganization of the actin cytoskeleton is essential for cancer cell migration and invasion, and for epithelial-mesenchymal transition (EMT), where epithelial cancer cells acquire more motile mesenchymal properties [27, 28]. Actin filaments are built of monomers that polymerize into a double-helix structure [26]. Cofilin is a key regulator of actin cytoskeleton, enhancing the severing of actin filaments and providing actin monomers for the polymerization of new filaments [29]. The cofilin pathway has been shown to be critically involved in the regulation of tumor cell migration and invasion [30–32]. Constitutively active cofilin advanced PC-3 cell invasion and lung metastasis *in vivo* in nude mice bearing PC-3 cell xenografts [33]. Besides its major role in modulation of actin dynamics and migration/invasion, cofilin has recently been shown to have many other cellular actions, such an involvement in induction of apoptosis and the maintenance of nuclear structure and functions [32]. In addition, mitochondrial translocation of cofilin was found to be involved in TGF beta-induced apoptosis of prostate cancer cells [33].

The activity of cofilin is regulated by phosphorylation-dephosphorylation reactions, interaction with phosphatidylinositol-4,5-bisphosphate at the plasma membrane or binding to cortactin. Phosphorylation of cofilin at Ser 3 by LIM or TES kinases inactivates the protein, and dephosphorylation at Ser 3 by SSH (slingshot) and some other phosphatases activates it, which reactions primarily determine cofilin regulation of actin dynamics [31, 32]. Besides modulation of cofilin protein and protein interactions, cofilin overexpression, associated with increased activity, has been reported in several cancers. In a prostate cancer patient cohort, cofilin levels were increased and they were significantly higher in metastases than in primary tumors [33]. Cofilin level was also increased in bladder cancer [34], and in ovarian [35] and pancreatic cancer [36] overexpression of cofilin was associated with poor prognosis. In non-small-cell lung cancer (NSCLC) high cofilin level correlated with poor outcome and cisplatin treatment resistance [37].

Of other proteins modulating actin cytoskeleton, p21-associated kinases (PAK) activate LIM kinase and have wide effects on cancer cell migration and invasion as well as proliferation and survival [27, 31, 38, 39]. Focal adhesion kinase (FAK), paxillin and integrins are focal adhesion-associated regulatory proteins which modulate actin dynamics and cytoskeletal organization in the initiation of cell migration [40]. In addition to ALN-induced changes in F-actin organization in prostate cancer cells [6, 13], zoledronate has been demonstrated to inhibit the expression of alphavbeta3 and alphavbeta5 integrins in endothelial cells [41] and to induce detachment of prostate cancer cells in association with FAK dephosphorylation [42].

In this study, we investigated the mechanisms by which ALN disrupts actin cytoskeleton organization and inhibits PC-3 cell invasion and migration. To achieve this, we used invasion and migration assays, F-actin stainings, and photobleaching (FRAP) technique to examine the kinetic fluorescence recovery. We also studied the effects of ALN on the levels of cofilin, PAKs, β1-integrin, paxillin and FAK by immunostainings, flow cytometry, and Western blotting. The role of cofilin in invasion, migration and F-actin organization was studied by silencing cofilin expression with siRNA and transfecting PC-3 cells with cofilin expressing vector. Our results demonstrate that ALN-induced inhibition of invasion/migration, and disruption of F-actin organization in prostate cancer cells were associated with markedly lowered levels of cofilin protein *in vitro*, and in an *in vivo* tumor model. Decrease of cofilin with siRNAs caused similar cellular effects as ALN, whereas transfection with cofilin expressing vector opposed ALN inhibition of cell migration. Our results suggest that the decrease of cofilin has an important role in ALN-mediated inhibition of prostate cancer invasion and metastasis.

## RESULTS

### ALN inhibits PC-3 cell invasion and migration in a time-dependent manner

**Figure 1:**
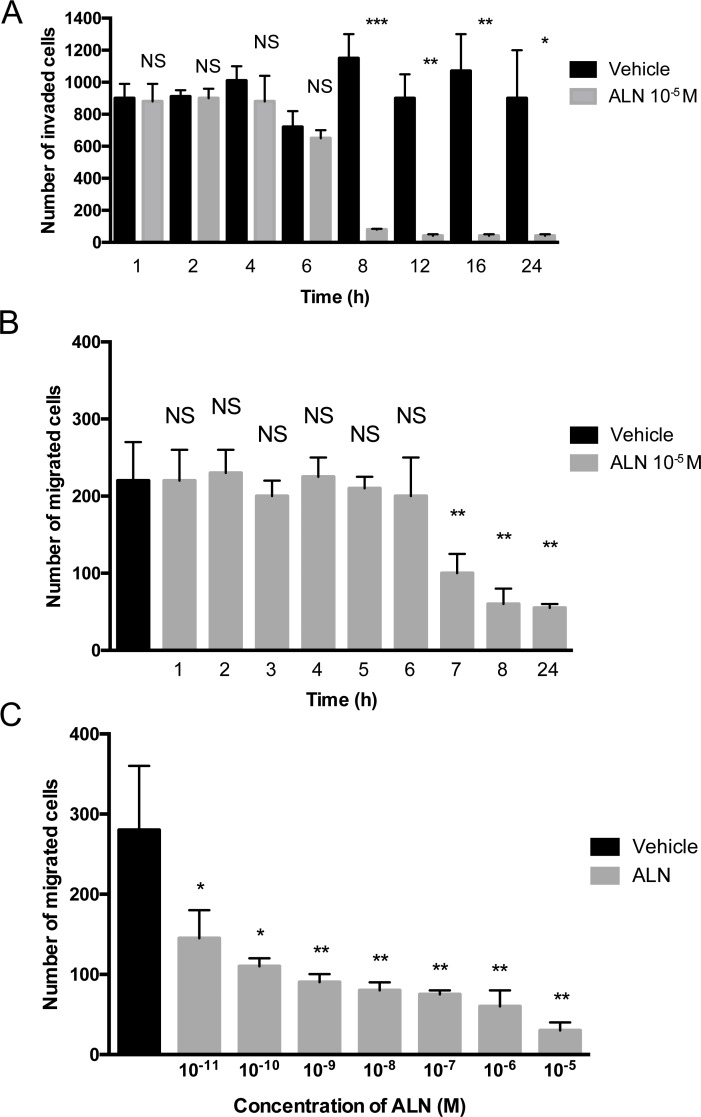
ALN decreases PC-3 cell invasion and migration in a Boyden chamber assay PC-3 cells were pretreated with 10^-5^ M ALN (gray bars) or 1% BSA-DMEM (black bars) for 1-24 hours (Time = pretreatment time) and then incubated in the absence of ALN in an invasion assay for 48 h **(A)** and in a migration assay for 5 h **(B)**, or the cells were pretreated with various ALN concentrations (10^-11^-10^-5^ M) for 24 h and then incubated in a migration assay for 5 hours in the absence of ALN **(C)**. The number of invading cells was counted as described in Materials and Methods. ^*^p< 0.05, ^**^p< 0.01, ^***^p< 0.001, NS = not significant versus control. The experiment was repeated 3 times.

To study the time course of the ALN effect on invasion, PC-3 cells were treated for various time periods (1-24 h) with 10^-5^ M ALN or vehicle, and incubated for 48 hours in *in vitro* invasion assays. Significant inhibition of invasion by ALN was achieved within 8 hours (*p*=0.0001) (Figure 1A). ALN also inhibited migration in a Boyden chamber assay, where the inhibitory effect was achieved within 7-8 hours (*p*=0.003) (Figure 1B). In comparison to vehicle, 50% inhibition to migration was achieved using 10^-11^M ALN concentration (Figure 1C).

### ALN disrupts actin organization and dynamics, and decreases PAK2 levels

**Figure 2:**
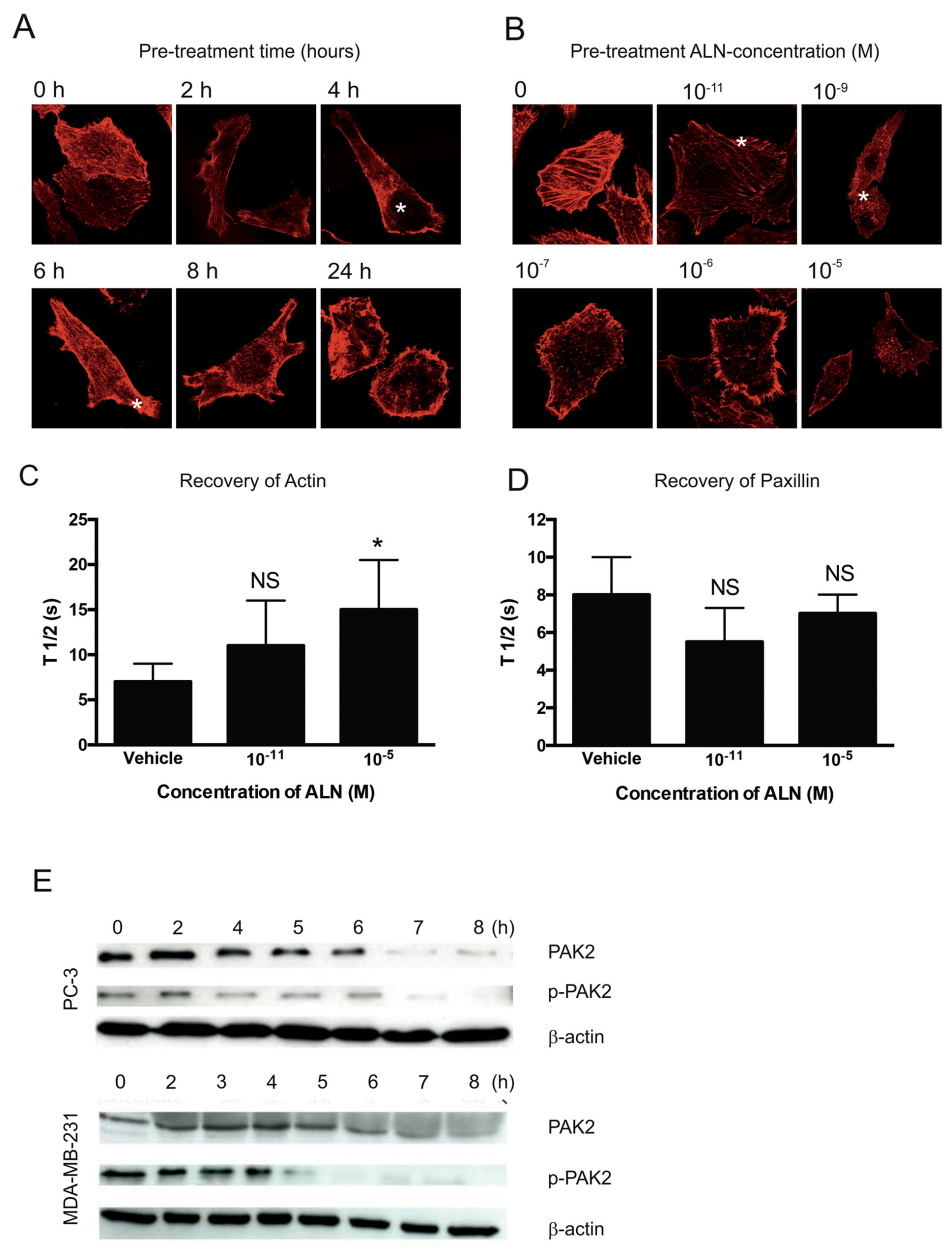
ALN interferes with F-actin organization in PC-3 cells PC-3 cells were pretreated with 10^-5^ M ALN for 2-24 hours and the cells were seeded at a concentration of 3000 cells/well on matrigel-coated round coverslips and allowed to adhere for 5 hours. The cells were then fixed and stained with phalloidin, prepared for microscopy, and imaged with a confocal microscope **(A)**. PC-3 cells were pre-treated with indicated concentrations of ALN for 8 hours and the cells were then seeded on matrigel-coated slides and allowed to attach for 5 hours. Cells were stained with phalloidin, prepared for microscopy and imaged with a confocal microscope. Stars indicate F-actin dysruption **(B)**. Effect of ALN treatment on the dynamics of actin **(C)** and paxillin **(D)** was studied in PC-3 cells transfected withGFP-actin or GFP-paxillin using FRAP (fluorescence recovery after photobleaching). The cells were treated with DMEM+1% BSA (vehicle), or 10^-11^M or 10^-5^ M ALN for 3 h. FRAP experiments were performed with Zeiss LSM510 META confocal microscope. The data were assessed by means of FCalc^®^, and half times of recovery (T½ values) were calculated. Each treatment was carried out in triplicate. ^*^p< 0.05, NS = not significant versus control. **(E)** PC-3 and MDA-MB-231 cells were treated for 2–8 hours with 10^-5^ M ALN. The levels of total PAK and p-PAK2 were detected by Western blotting. The levels of total PAK and p-PAK2 decreased 5-6 hours after ALN-treatment in both cell lines used. The experiments were repeated 3 times.

The time course and concentration-dependency of ALN on F-actin organization was studied in PC-3 cells. Cells were treated for various time periods with 10^-5^ M ALN, and F-actin was stained with phalloidin. F-actin disruption was achieved 8 hours after ALN addition, but minor effects were already seen within 4-6 hours (Figure 2A, stars). The experiments with 10^-11^ to 10^-5^ M ALN concentrations showed that minor disruption of F-actin was observed with 10^-11^ M ALN, and total disruption of F-actin organization was achieved with an 8-hour treatment in the presence of 10^-9^ M ALN (Figure 2B, stars). Corresponding concentration-dependent effects were also seen in ALN-treated DU-145 cells (data not shown). The effect of ALN on actin and paxillin dynamics was studied using fluorescence recovery after photobleaching (FRAP) with GFP-actin or GFP-paxillin-transfected PC-3 cells. 48 hours after transfection, PC-3 cells were treated with 10^-11^ or 10^-5^ M ALN for 3 hours. A region of interest containing visible actin or paxillin structures was photobleached with a 488 nm laser, and fluorescence recovery was monitored. Half-time of recovery (T_1/2_) values were assessed. 10^-5^ M ALN (*p*= 0.023) but not 10^-11^ M ALN (*p*=0.067) inhibited actin recovery, compared with vehicle treatment (Figure 2C). ALN did not have any effect on paxillin recovery at either concentration (*p*= 0.09 and *p*= 0.672, respectively) (Figure 2D). Total PAK and phosphorylated PAK2 (p-PAK2) levels were decreased in 4-7 hours after addition of ALN to PC-3 and MDA-MB-231 cells (Figure 2E), but ALN had no effect on FAK, paxillin or β1-integrin levels (data not shown).

### ALN decreases cofilin levels *in vitro*

**Figure 3:**
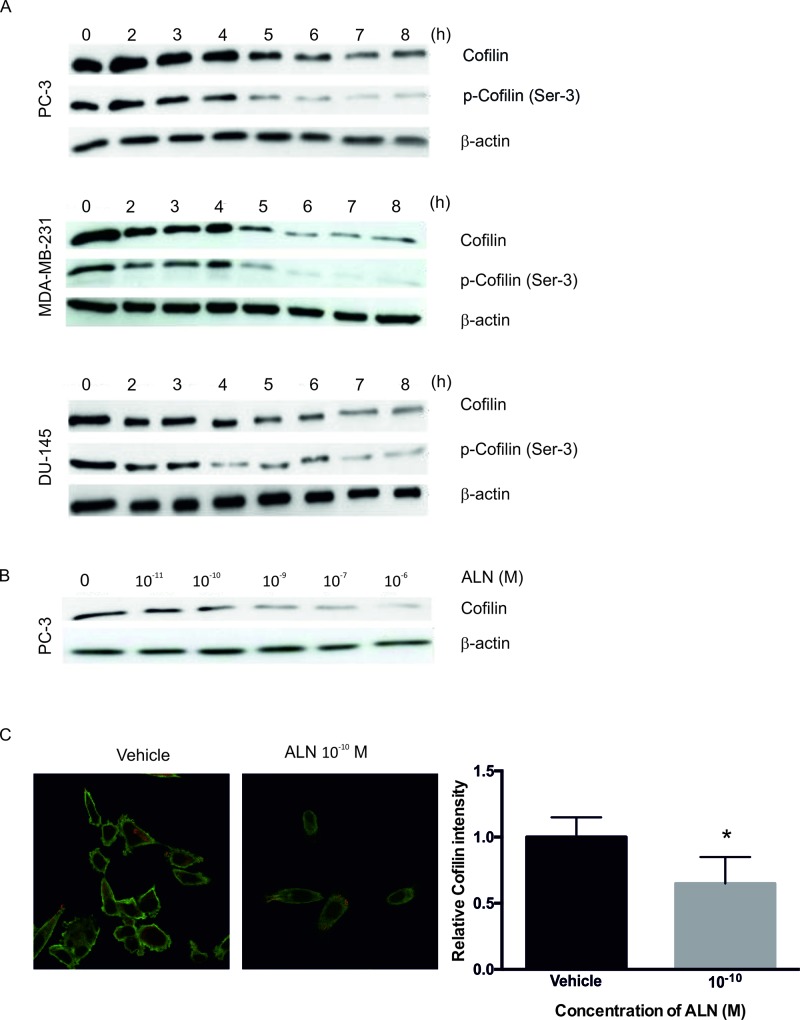
ALN decreases cofilin protein levels in PC-3, DU-145 and MDA-MB-231 cells Cells were treated with 1% BSA-DMEM (vehicle) or 10^-5^ M ALN for 2-8 hours **(A)**. PC-3 cells were treated with 10^-11^ to 10^-5^ M ALN for 8 hours to study the effect on the level of total cofilin **(B)**. The experiments were repeated 3 times. Cofilin immunostaining intensity was analyzed from control and 10^-10^ M ALN-treated cells **(C)**. ^*^p< 0.05 versus vehicle.

The effects of ALN on the levels of actin-related cytoskeletal proteins in PC-3, DU-145 and MDA-MB-231 cells were studied by Western blotting. Decrease in the levels of phosphorylated cofilin (p-cofilin) as well as total cofilin was seen in all three cell lines. Decreases in cofilin levels with 10^-5^ M ALN appeared in 4-7 hours (Figure 3A), and even at very low ALN concentrations (10^-9^ to 10^-10^ M) in PC-3 cells (Figure 3B). Cofilin staining intensity in PC-3 cells treated with 10^-10^ M ALN was significantly reduced compared with control treatment (*p*=0.026) (Figure 3C). The effect of ALN on cofilin mRNA level was examined by means of qRT-PCR. No effects were seen at the ALN concentrations used (10^-8^-10^-5^ M) at 8 hours (Supplementary Figure 1). Similar results were observed in DU-145 and VCaP cells (data not shown). Clodronate had no effect on the level of total or phosphorylated cofilin (data not shown).

### ALN decreases cofilin levels in PC-3 tumors

**Figure 4:**
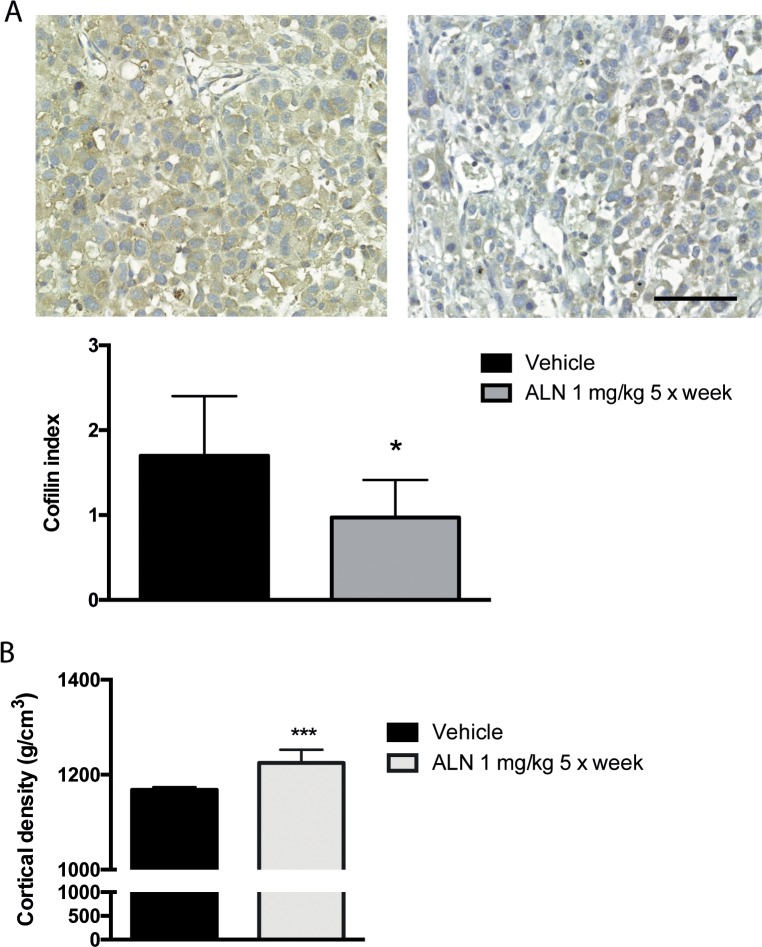
ALN decreases cofilin level of *s.c*. PC-3 tumor xenografts Nude mice were injected *s.c*. with 10^6^ PC-3 cells and treated with *i.p.* injections of ALN (1 mg/kg, 5 days/week) for 4 weeks before sacrifice. Cofilin index was decreased in PC-3 *s.c.* tumors of the nude mice treated with ALN compared with tumors of vehicle-treated mice. Cofilin index was calculated by multiplying staining intensity with percentage of cofilin-positive cells. Bar represents 100 μm, ^*^p< 0.05 versus vehicle **(A)**. pQCT analysis shows that bone mineral density increased in ALN-treated mice. The measurement was done as an intrinsic control for ALN efficacy and biological activity **(B)**.

In order to detect ALN effects on cofilin *in vivo*, subcutaneous tumors were produced. Mice were treated with vehicle or ALN (1 mg/kg) five times a week. After five weeks, mice were sacrificed and tumors were analyzed. Immunohistochemical cofilin stainings were scored by multiplying the percentage of cofilin-positive tumor cells, and staining intensity. Results were shown as cofilin index. Corresponding to the results with PC-3 cells, decreased cofilin staining level was also seen in ALN-treated subcutaneous PC-3 tumors (Figure 4A), *n*= 16 + 16, *p* < 0.05, Mann-Whitney test. ALN reduced slightly the volume of PC-3 xenografts but the result was not statistically significant (data not shown). Metastasis could not be studied in this model, since PC-3 cells do not invade through the tumor capsule in subcutaneous xenografts. Effects on angiogenesis and proliferation were investigated using immunohistochemical stainings of tumor sections with CD34 and pH3 antibodies, respectively. There was no difference between groups (data not shown). The functionality of ALN was shown by increased bone mineral density in ALN-treated tumor bearing nude mice (Figure 4B). ALN had no effect on mouse body weights (data not shown).

### Cofilin knockdown disrupts invasion and F-actin organization in PC-3 cells

**Figure 5:**
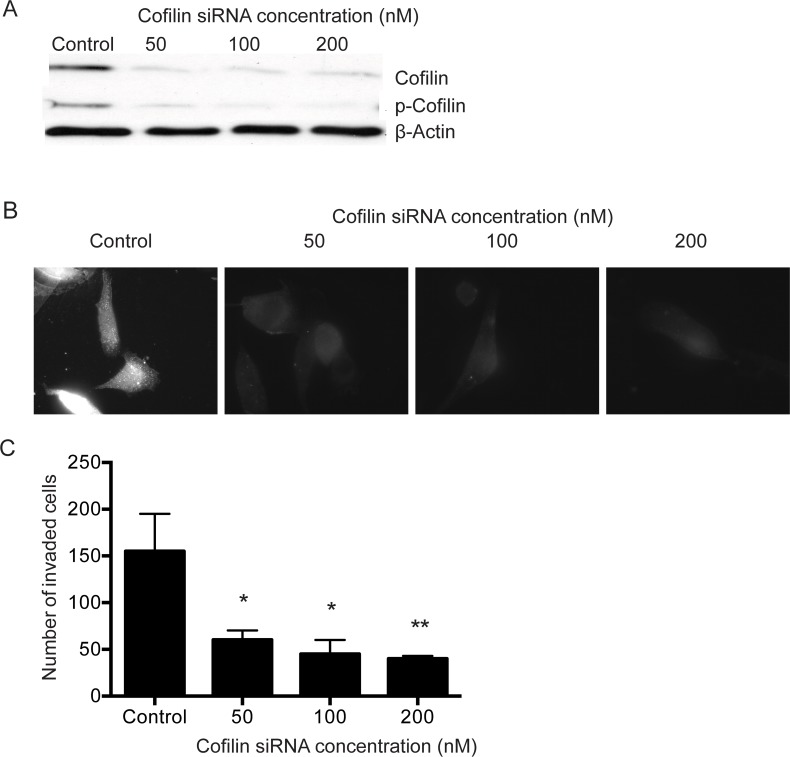
Knock-down of cofilin using siRNA disrupts F-actin organization and inhibits invasion of PC-3 cells PC-3 cells were seeded on 6-well plates or glass slides and transfected with 50, 100 and 200 nM cofilin 1 siRNA or with control siRNA as described in Materials and Methods. After 60 hours, the cells were lysed for Western blotting to detect the levels of total and p-cofilin **(A)**, fixed on glass slides and immunostained for cofilin **(B)**, or detached and incubated for 48 hours in an invasion assay **(C)**. After invasion assay, cells were fixed, stained and counted as described in Materials and Methods. ^*^p< 0.05, ^**^p< 0.01 versus control. The experiments were repeated 3 times.

To study the role of cofilin in PC-3 cell function, siRNA silencing experiments were performed. Transfection of PC-3 cells with cofilin siRNA (50, 100 and 200 nM) clearly decreased the levels of total and p-cofilin protein, when compared with control siRNA-transfected cells, (Figure 5A). Immunostaining of PC-3 cells transfected with 50, 100 or 200 nM cofilin siRNA also showed that cofilin levels were effectively decreased (Figure 5B). F-actin organization was disturbed in cofilin-depleted cells studied by phalloidin staining. Cofilin siRNA (100 and 200 nM) disrupted actin stress fibers and induced actin aggregates in stress fibers (Supplementary Figure 2) and significantly inhibited PC-3 cell invasion at concentrations 50, 100 and 200 nM (*p=*0.020, *p=* 0.013, *p=*0.009, respectively) (Figure 5C).

### Transfection of PC-3 cells with a cofilin expressing vector is associated with decreased response of cellular cofilin level and migration to ALN treatment

**Figure 6:**
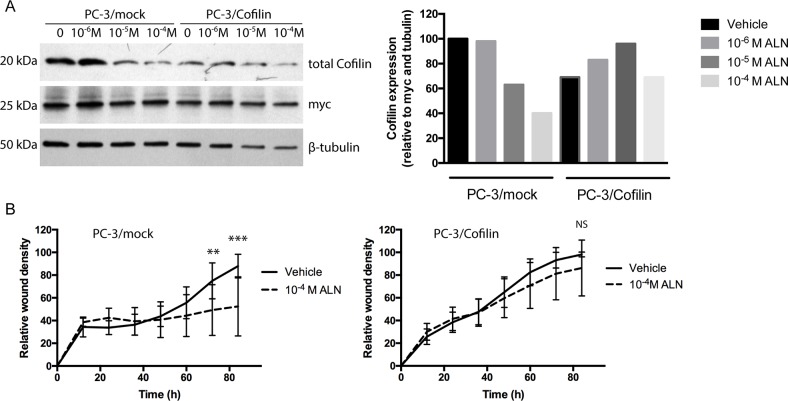
Transfection of PC-3 cells with a cofilin expressing vector makes PC-3/cofilin cells more resistant to ALN treatment PC-3 cells were transiently transfected with a cofilin expression vector including human myc as a tag gene (PC-3/cofilin cells) or with a control vector (PC-3/mock cells). The cells were treated with 10^-4^-10^-6^M ALN for 24 h and analyzed with Western blots for levels of total cofilin **(A)**. Success of transfection and loading of protein were assessed on stripped blots by immunodetection of myc (as a transfection control) and tubulin (as a loading control). Results are shown as relative cofilin level (normalized to tubulin and myc). PC-3/mock and PC-3/cofilin cells were pretreated with 10^-4^M ALN for 24 h. Then, medium was changed to 1%BSA-DMEM and migration was studied in a wound density assay up to 92 h **(B)**. ALN inhibited migration of PC-3/mock cells but did not affect the migration of PC-3/cofilin cells. Results are expressed as mean values together with standard deviation. Mean changes over time were compared with hierarchical linear mixed models suitable for repeated measures. In addition, differences of model based means were compared at each time point and they are shown in four time points (24h, 48h, 72h and 92h).

Next, we transiently transfected PC-3 cells with a cofilin expression vector containing human myc as a tag gene. Expression of the tag myc was clear in the cells transfected either with the construct bearing cofilin gene (PC-3/cofilin cells) or with the control construct lacking cofilin (PC-3/mock) suggesting that transfections and the functions of the vectors were successful (Figure 6A). Unexpectedly, however, in repeated experiments the level of total cofilin was not generally increased in transfected PC-3/cofilin cells compared with mock-transfected control cells (PC-3/mock). Transfection efficiency was approximately 40 % (data not show). Nevertheless, ALN did not decrease cofilin levels in PC-3/cofilin cells to the same degree as in PC-3/mock cells (Figure 6A) suggesting that the cells transfected with cofilin expressing vector were able to maintain sustained, even if not increased level of total cellular cofilin, which was associated with increased resistance to ALN treatment. Concomitantly, migration of PC-3/cofilin cells measured by a scratch wound assay was not inhibited by a 24-h ALN (10^-4^ M) pretreatment whereas as in PC-3/mock cells ALN pretreatment did decrease migration when compared to vehicle-treated cells during a 92-hour assay (Figure 6B, 72h *p*< 0.01, 92h *p*< 0.001).

## DISCUSSION

In addition to various osteoclast-specific effects, we and others have shown that the N-BP ALN inhibits adhesion and invasion of various cancer cell lines and that it has anti-tumoral effects *in vivo* [11, 17, 24, 43]. ALN causes disruption of actin cytoskeleton [6] and reduces the activity of small GTPases, which are important in actin polymerization and organization of the cytoskeleton [25, 44]. Our previous study showed that ALN also inhibited PC-3 cell invasion even at low concentrations [13]. This finding aroused our interest in studying further the effects of ALN on invasion, migration, and the proteins regulating cytoskeleton.

The appropriate actin dynamics is essential for cell motility [25, 26]. We saw visible changes in F-actin organization 4-6 hours after addition of ALN (10^-5^ M) to PC-3 prostate cancer cell cultures. In addition, in the FRAP analysis of GFP-actin or GFP-paxillin-transfected PC-3 cells, the recovery time of GFP-actin, but not that of GFP-paxillin, was clearly prolonged in the cells treated with ALN for 3 hours. Visible disruption of F-actin was also seen at lower ALN concentrations (10^-11^ to 10^-9^ M) in PC-3 cells and in DU-145 cells (data not shown) 4-6 hours after ALN addition. It is plausible that ALN has minor, non-detectable but crucial effects on actin even at lower concentrations. These results suggest that the regulation of actin cytoskeleton is involved in ALN effects on invasion and migration.

ALN impairment of actin organization/dynamics and inhibition of invasion were associated with a strong decrease of cofilin level in PC-3 cells. Both total and phosphorylated cofilin were lowered. Corresponding effects were also seen in DU-145 prostate cancer cells and MDA-MB-231 breast cancer cells. Similar to ALN treatment, depletion of cofilin with siRNAs caused a strong inhibition of PC-3 invasion. Our results are in line with others [31, 45] which have previously shown that cofilin suppression via siRNAs disrupts motility and actin organization in mammalian cells. ALN treatment and siRNA depletion of cofilin thus seemed to affect cellular cytoskeleton and motility similarly suggesting that decrease of cofilin has a central role in ALN effects. This conclusion is also supported by our result that ALN could not decrease total cellular cofilin level in and migration of PC-3 cells transfected with cofilin expressing vector to the same extent as of the cells transfected with a mock control vector.

Besides cofilin, ALN decreased total and phosphorylated PAK2. The PAK proteins have important roles in regulation of the cofilin pathway and actin cytoskeleton as well as other cellular functions including proliferation and apoptosis [46]. Interestingly, elevated levels of PAK2 were found in phosphoproteomic analysis of castration resistant prostate cancer and knockdown experiments demonstrated that PAK2 regulates prostate cancer cell invasion [47]. PAK2 is located upstream of cofilin and it may thus contribute to ALN effect on cell invasion/migration which deserves further studies. Other attachment, cytoskeleton and cell motility-regulating proteins, which were studied (FAK, β1-integrin, paxillin or dynamics of paxillin) were not altered. Therefore, the inhibitory effect of ALN on cell migration seemed to be associated with actin- and cofilin-related regulatory pathways rather than focal adhesion-related proteins.

Cofilin overexpression has been seen in lung cancer cells undergoing EMT [48] and in association with increased tumor cell invasion or metastasis [34, 49–51]. Collazo and coworkers reported augmented levels of active cofilin in human prostate cancer and in experimental prostate tumors [33]. They also showed that decreasing cofilin expression by gene silencing methods decreased cancer cell invasion/migration and tumor metastasis [33]. These results suggest that cofilin regulation of invasion and migration in cancer cells is not only dependent on cofilin activity, largely determined by the phosphorylation status of the protein, but also by the level of cofilin expression.

Our present results show that similar to cofilin knockdown with siRNAs, the bisphosphonate ALN-decreased invasion/migration was associated with decreased cofilin in prostate cancer cells. Our results further demonstrate that transfection of PC-3 prostate cancer cells with cofilin expression vector not only opposed ALN inhibition of migration of the cells but also interfered with ALN reduction of cofilin. Interestingly, transfection did not generally lead to increased cofilin levels in the cells although expression of the myc-tag gene in transfected cells suggested that the construct was functional and transfection successful. Nevertheless, the results demonstrate that the ALN effect on prostate cancer cell migration was blocked in cofilin-transfected cells. Although the mechanisms remain unclear it is possible that instead of increased level for example altered turnover rate of cofilin might have been able to block ALN inhibition of migration in transfected cells. The time lag of several hours between ALN administration and cofilin decrease accompanied by inhibition of invasion/migration suggests that ALN effects are indirect and that either *de novo* protein synthesis and/or changes in protein degradation would be required. ALN treatment did not, however, alter the levels of cofilin mRNA. One possibility is that the well-known inhibitory effects of ALN and other N-BPs on the mevalonate pathway causing deficient isoprenylation and impaired function of small regulatory GTPases could lead to a decrease in cofilin levels. This possibility is supported by our previous results demonstrating that treatment of PC-3 cells with specific inhibitors of FTase (farnesyltransferase) and GGTase (geranylgeranyltransferase) that block isoprenylation reactions, similarly caused strong depletion of both phosphorylated and total cofilin [6]. It is conceivable that ALN inhibition of prenylation of upstream regulatory proteins might lead to altered turnover of cofilin (and PAK) proteins by the mechanisms that remain to be clarified.

Besides PC-3 cells *in vitro*, decrease of cofilin was seen in PC-3 tumors of ALN-treated nude mice. We have previously shown in an orthotopic prostate cancer mouse model that ALN decreased metastasis to prostate-draining iliac and sacral lymph nodes. ALN treatment also affected orthotopic tumor growth which in that study was found to be associated with decreased vascularization and increased apoptosis [17]. Recent studies suggest that cofilin is involved in many cellular functions other than regulation of actin dynamics and cellular motility only [32], but whether they have any role in ALN effects on tumor growth is presently unclear.

Although our results demonstrate that ALN can affect cofilin and cell invasion/migration *in vitro* at very low concentrations and that systemic ALN treatment can decrease cofilin level in xenografts and also growth rate and metastasis in an orthotopic tumor model [17], therapeutic exploitation of effects of ALN and possible other N-BP on prostate tumors might be a challenge. It has been shown that the N-BP zoledronate accumulates transiently in the prostate before entering the bone *in vivo* [52], but still all BPs rapidly bind to bone hydroxyapatite with 99% efficiency and less than 1% of administered BP remains in the blood circulation [53]. This emphasizes the importance of continuous N-BP dosing in the treatment of cancer cells/tumors in order to maintain the inhibitory effects of N-BPs. Alternatively, the N-BP bioavailability could possibly be extended for example encapsulation into nanocarriers, which could be targeted to tumors [54–56].

Taken together, our results suggest that down-regulation of cofilin protein is important for ALN-induced disruption of actin cytoskeleton and inhibition of prostate cancer cell invasion and migration. The results emphasize the potential of ALN and other N-BPs in the inhibition of prostate cancer invasion and metastasis.

## MATERIALS AND METHODS

### Cell lines and production of conditioned medium

The cells lines used were PC-3 and DU-145 (androgen-independent human prostate carcinoma cell lines; ATCC, USA), VCaP (androgen-sensitive human prostate carcinoma cell line, ATCC, USA), MDA-MB-231 (SA) (triple-negative human breast carcinoma cell line; a kind gift from Dr. Theresa Guise, San Antonio), and MG-63 (an osteosarcoma cell line; ATCC, USA). All cell lines were cultured in DMEM (Gibco, USA), containing heat-inactivated fetal bovine serum (iFBS; 10%) (Gibco, USA) at 37°C in a humidified atmosphere (5% CO_2_). All *in vitro* experiments were performed in DMEM containing 1% BSA (Sigma, USA). For conditioned medium, MG-63 cells were cultured for 10 days in DMEM containing iFBS (10%) and ascorbic acid (0.5 μg/ml) (E. Merck, Germany), and then for 2 days in DMEM containing BSA (0.1%) and ascorbic acid (0.5 μg/ml). Conditioned medium was collected from confluent cultures, centrifuged and frozen. For *in vivo* experiments, the cells were harvested at near confluence and suspended at a concentration of 10^6^ / 100 μl in phosphate-buffered saline (PBS; Biochrom AG, Berlin, Germany). The cells were kept on ice until inoculation. Cell viability was over 95% at the time of inoculation, as determined by trypan blue staining of the cell suspension.

### Cofilin transfection and RNAi knockdown

For cofilin transfection, PC-3 cells were seeded on 6-well plates. When the cells reached 70% confluency, they were transfected with the expression vector pPL108-EGFP containing human cofilin and human myc as a tag gene, (PC-3/cofilin cells) or with an empty vector (PC-3/mock cells) (Vartiainen et al., 2002). The transfection was carried out using FuGENE^®^HD Transfection reagent (Promega Corporation, Madison, WI, USA) according to the manufacturer's instructions. Transfection efficiency was confirmed by GFP signal, which was seen in approximately 40% of the cells with the Evos FL microscope (ThermoFisher Scientific, Waltham, MA, USA) (data not shown). For cofilin knockdown, PC-3 cells were seeded on 6-well plates and allowed to grow to 50% confluence in 10% iFBS-DMEM. The cells were then transfected with 50–200 nM human cofilin 1 siRNA (Santa Cruz Biotechnology, USA) or with control siRNA (Santa Cruz Biotechnology, USA) by Oligofectamine (Invitrogen, USA) in OptiMEM (Gibco, USA). Transfections were performed according to the manufacturer's instructions. After 60 hours, the cells were prepared for further experiments.

### Chemical compounds

Alendronate (4-amino-1-hydroxybutylidene-1.1-bisphosphonic acid) was kindly provided by Merck, Sharp & Dohme (West Point, PA, USA) and clodronate (dichloro-methylene bisphosphonic acid) was from Leiras (Turku, Finland).

### Invasion assays

PC-3 cells were treated with 10^-5^ M ALN or 1% BSA-DMEM (as a control) for 1-24 hours. Commercial cell culture invasion inserts of 8 μm pore size (Becton Dickinson, USA) were coated for invasion assays with Matrigel (30 μg/insert = 100 μg/cm^2^; Becton Dickinson, USA) for 24 hours to prepare an *in vitro* basement membrane. Assay was started by adding 5 × 10^4^ cells in 300 μl of DMEM + 1% BSA to the upper chamber and 300 μl of DMEM + 1% BSA plus 300 μl of MG-63-conditioned medium in the lower chamber as a chemoattractant. Cells were incubated for 48 hours at 37 °C and in 5% CO_2_, and the insert membranes were prepared for microscopy. The membranes were first fixed for 10 minutes in 4% paraformaldehyde in PBS (J.T. Backer, Holland) and stained with Mayer's haematoxylin (Zymed, USA) for 24 hours. After washing, the membranes were cut from the inserts, the cells on the upper surface of the membrane were wiped off with a cotton wool bud and the membranes were mounted with glycerol-PBS (9:1, E. Merck, Germany). The number of cells on the lower surface of the membrane was counted under microscope (10x objective) from 10 consecutive fields, representing 40% of the total area of the membrane. The experiments were repeated three times and each treatment was carried out in triplicate.

### Boyden chamber and scratch wound migration assays

For Boyden chamber migration assay, PC-3 cells were pre-treated with 1% BSA-DMEM (as a control) for 24 h, 10^-5^ M ALN for 1-24 hours or with various ALN concentrations (0-10^-5^M) for 1 to 24 hours. Commercial cell culture invasion inserts of 8 μm pore size (Becton Dickinson, USA) were coated with laminin (5 μg/cm^2^; Becton Dickinson, USA). Assay was started by adding 5 × 10^4^ cells in 300 μl of DMEM + 1% BSA to the upper chamber and 300 μl of DMEM + 1% BSA plus 300 μl of MG-63-conditioned medium in the lower chamber as a chemoattractant. Cells were incubated for 5 hours at 37 °C and in 5% CO_2_, and the insert membranes were prepared for microscopy similarly to invasion assay. The experiments were repeated three times and each treatment was carried out in triplicate. For scratch wound cell migration assay, 4 × 10^4^ PC-3, PC-3/mock or PC-3/cofilin cells were seeded into 96-well ImageLock plates (Essen BioScience, Ann Arbor, MI, USA). When the wells were confluent, the cells were treated with 10^-8^-10^-4^ M ALN or 1% BSA-DMEM (vehicle) for 24 hours. Scratch wounds were made using a 96-pin WoundMaker tool (Essen BioScience) and medium was changed to 1% BSA-DMEM without ALN. Plates were imaged and wound areas were automatically calculated using the IncuCyte ZOOM kinetic imaging platform and IncuCyte ZOOM 2014A software (Essen BioScience). The experiment was terminated when the vehicle-treated wounds reached 100% confluency. The results are shown as relative wound confluency.

### Fluorescence staining

PC-3 cells were pre-treated with various ALN concentrations for 8 hours, or for various time periods with 10^-5^ M ALN or control (DMEM containing 1% BSA) for 24 hours. After 5 hours adhesion on coverslips coated with Matrigel (Becton Dickinson, USA), adhering cells were fixed with 4% PFA, and permeabilized with 0.5% Triton X-100 (Sigma, ST Louis, Missouri, USA) for 5 minutes. For F-Actin visualization, cells were stained for 20 minutes with TRITC-labelled phalloidin (0.2 μg/ml) (Sigma, USA). Immunocytochemical cofilin staining was performed in 3% PFA-fixed cells by blocking with 0.1% BSA in PBS for 1 hour and then incubating with anti-cofilin antibody (Abcam, USA) for an additional hour. After PBS washings, cells were stained with chicken anti-rabbit IgG (H+L) Alexa Fluor^®^ 488 (Invitrogen, USA) and TRITC-phalloidin (0.2 μg/ml) (Sigma, USA) for 1 hour. After PBS washes, the coverslips were mounted, and images were acquired with Zeiss LSM510 META confocal microscope (Zeiss GmBH, Jena, Germany). Hoechst 33342, Alexa Fluor 488 and TRITC-phalloidin were excited at 405 nm, 488 nm and 543 nm laser lines, and emissions were collected via 420–480 nm, 500–550 nm and 560 LP (longpass) filters, respectively. The cofilin staining intensities were analyzed using ImageJ 1.42 software [57].

### Fluorescence recovery after photobleaching (FRAP)

PC-3 cells were transfected with pEGFP-Actin (Clontech, Palo Alto, CA, USA) or pEGFP-Paxillin (kind gifts from Dr Eleanor Coffey, Turku Centre for Biotechnology, Finland), using 3 μg vector DNA and 7 μl TransFectin Lipid Reagent (Bio-Rad, USA) in glass-bottom cell culture dishes (MatTek, MA, USA). The cells were incubated for 5 hours and then the medium was changed. The cells were cultured for an additional 48 hours to achieve GFP expression. Transfected cells were treated for 3 hours with either DMEM + 1% BSA (negative control), or 10^-11^ or 10^-5^ ALN. FRAP experiments [reviewed in [58]] were performed with Zeiss LSM510 META confocal microscope in a humidified chamberat 37 °C and 5% CO_2_. Cells transiently expressing GFP-actin or GFP-paxillin were excited with a 488 nm laser beam and emission was collected via the 500–550 nm bandpass filter. Prior to photobleaching, three images were collected. A region of interest (ROI) was chosen and it was photobleached (488 nm; 100% intensity). Recovery was followed at 2-second intervals. The half time of recovery (T½) and the mobile fraction (M_f_) were calculated. The data were assessed by means of FCalc^®^ (Rolf Sara, Turku Centre for Biotechnology, Finland). Briefly, acquired data is corrected for image acquisition-caused photobleaching and the resulting data is fitted to the equation y = (1-exp(kt)).

### Western blotting

Cells were cultured until semi-confluent in tissue culture dishes, and were treated with various ALN concentrations for 8 hours, or for various time periods with 10^-5^ M ALN or 1% BSA-DMEM. The cells were lysed in standard Laemmli sample buffer with β-mercaptoethanol and aliquots were boiled for 5 minutes at 100°C. 30 μl of whole-cell lysate/treatment were subjected to SDS-PAGE with molecular weight standards (Bio-Rad, USA) and transferred to nitrocellulose membranes (Millipore, MA, USA). The membranes were blocked with 8% skimmed milk in Tris-buffered saline with 0.05% Tween 20 (MP Biomedicals, Solon, Ohio, USA). After 1 hour of blocking, the membranes were incubated with primary antibodies against total cofilin and phospho-cofilin (Ser-3) (Cell Signaling Technology, USA), total paxillin and phospho-paxillin (Tyr118) (Cell Signaling Technology, USA), total FAK and phospho-FAK (pY397) (BD Bioscience, USA), total PAK and phospho-PAK2 (p-PAK2) (Ser141) (Cell Signaling Technology, USA), and c-Myc-tag (Invitrogen, USA). Loading of protein was assessed on stripped blots by immunodetection of β-Actin (Sigma, USA) or tubulin (Abcam, Cambridge, UK). After washing with TBS-1% Tween, the membranes were incubated with appropriate secondary antibodies. The proteins were visualized by enhanced chemiluminescence (Amersham Pharmacia Biotech, Sweden). Intensity of bands was analyzed using ImageJ (1.37v, Wayne Rasband, National Institute of Health, Bethesda, MD, USA).

### Flow cytometry

PC-3 cells were treated for 8 hours with 10^-5^ M ALN or 1% BSA-DMEM (as a control) and then detached and washed twice with staining buffer (1 ml PBS containing 2% FCS and 0.02% NaN_3_). The cells were stained at 4°C for 30 min in a final volume of 100 μl staining buffer containing saturating concentrations of monoclonal antibody (mAb) against β1-integrin or isotype-specific mAb 3G6 (BD Biosciences, USA). After washing with staining buffer, the cells were stained with FITC-conjugated anti-mouse Ig (Dako, Denmark). 10^4^ living cells were selected by forward and side scatter gating. Data were collected using FACSCalibur flow cytometer and analyzed with CellQuest Pro software (BD Biosciences, USA).

### Quantitative RT-PCR

ALN-treated PC-3 cells were lysed and total RNA was purified by using an RNeasy Mini Kit (Qiagen). Complementary DNA was synthesized by using 1 μg of total RNA as starting material. Cofilin mRNA quantification was performed by QuantiTect SYBR green real time PCR kit (Qiagen) with a DNA Engine Opticon system (MJ Research, Inc., USA). Amplification conditions were as recommended in the Quantitect SYBR green handbook for two-step qRT-PCR (Qiagen, USA). The primers used were as follows: human cofilin: 5’-GATAAGGA CTGCCGCTATGC-3’, 5’-GCTTGATCCCTGTC AGCTTC-3’, human β-actin: 5’-CGTGGGG CGCCCCAGGCACCA-3, 5’-TTGGCCTTGGGGT TCA GGGGG-3’. Annealing temperature of 60 °C and 35 amplification cycles were used. The amount of cofilin mRNA was normalized to β-actin expression and each treatment was carried out in triplicate. The results were analyzed using the 2^-ΔΔCT^ method [59].

### Animal experiment

Mice were six-week-old in the beginning of the experiment. Male Hsd: Athymic Foxn1^nu^ mice (Harlan, the Netherlands) were maintained in a pathogen-free environment, under controlled conditions (20-21°C, 30-60% relative humidity, and a 12-hour light cycle). Animals were cared for in accordance with the Directives 2012/707/EU, 2014/11/EU and of the European Parliament and of the Council for the Care and Use of Laboratory Animals. Permissions were obtained from the State Provincial Offices of Finland (ESAVI/3257/04.10.07/2014). PC-3 cells (10^6^/100μl PBS) were inoculated subcutaneously into necks of mice. After tumors became palpable, mice were stratified into vehicle and ALN groups, according to the tumor volume (n= 8 + 8 mice). Mice were treated five times a week with vehicle (PBS) or ALN (1mg/kg, *i.p.*). Tumors were measured and mice were weighed 3 times a week. Mice were sacrificed at day 28. The experiment was repeated once. Tumors (n= 32, 16 PBS-treated and 16 ALN-treated) were collected for immunohistochemistry, and tibiae were collected for pQCT analysis.

### Immunohistochemical staining

PBS or ALN-treated PC-3 *s.c*. tumors were formalin-fixed, embedded in paraffin and cut into 5 μm-thick sections. The sections were stained with polyclonal rabbit anti-phospho-Histone-3 (Cell Signaling Technology, Danvers, MA, USA), monoclonal rat anti-CD34 (Santa Cruz Biotechnology Inc., Santa Cruz, CA) and polyclonal rabbit anti-cofilin antibody (Abcam, Cambridge, MA, USA) o/n at +4°C, followed by biotin-labeled anti-rat IgG (DAKO Denmark A/S, Glostrup, Denmark), or anti-rabbit IgG secondary antibodies (Sigma-Aldrich, St. Lois, MO, USA). Stained tumors were scanned using Pannoramic slide scanner (3DHistech, Budapest, Hungary). The number of pH3-positive cells and CD34-positive blood vessels were counted from whole tumor area using Pannoramic slide viewer (3DHistech, Budapest, Hungary) or ImageJ 1.48m software [57]. The percentage of cofilin-positive tumor cells was counted, and staining intensity was described as weak, moderate, or strong. Each tumor was scored (range 0-300) by multiplying the average intensity value by the average percentage of positively stained cells.

### pQCT analysis of bone mineral density

Bone mineral density was measured and analyzed from proximal tibia of tumor-bearing mice using peripheral quantitative computed tomography (Stratec XCT Research pQCT device, Norland Stratec Medizintechnik GmbH, Birkenfeld, Germany). The samples were measured using a voxel size of 0.07×0.07×0.5mm. For metaphyseal analysis of the tibiae, the scan lines were adjusted to 1-4 mm with 0.5 mm intervals, using the scout view given by the pQCT device. Calculations of cortical thickness were made using the ring model supplied by the Stratec software version 6.

### Statistical analyses

Statistical analyses were carried out using SAS® System version 9.4 for Windows (SAS Institute Inc., Cary, NC, USA). P values less than 0.05 (two-tailed) were considered as statistically significant. Normality of groups was tested by using Shapiro-Wilk's *W*-test and differences were tested for significance by using the Mann-Whitney U-test for independent samples.

## SUPPLEMENTARY MATERIALS FIGURES



## Abbreviations

BP: bisphosphonates
ALN: alendronate
EMT: epithelial-mesenchymal transition
FAK: focal adhesion kinase
FRAP: fluorescence recovery after photobleaching
FTase: farnesyltransferase
GGTase: geranylgeranyltransferase
iFBS: heat-inactivated fetal bovine serum
i.p: intraperitoneal
N-BP: Nitrogen-containing bisphosphonate
PAK: p21-associated kinase 2
s.c.: subcutaneous.
