# Supplementary material for: Alendronate-induced disruption of actin cytoskeleton and inhibition of migration/invasion are associated with cofilin downregulation in PC-3 prostate cancer cells
Source: Oncotarget. 2018 Aug 24;9(66):32593–608. doi: 10.18632/oncotarget.25961 (PMC6135693; doi:10.18632/oncotarget.25961)
Supplement: Supplementary file 1 [file oncotarget-09-32593-s001.pdf]

Alendronate-induced disruption of actin cytoskeleton and inhibition of migration/invasion are associated with cofilin downregulation in PC-3 prostate cancer cells

SUPPLEMENTARY MATERIALS

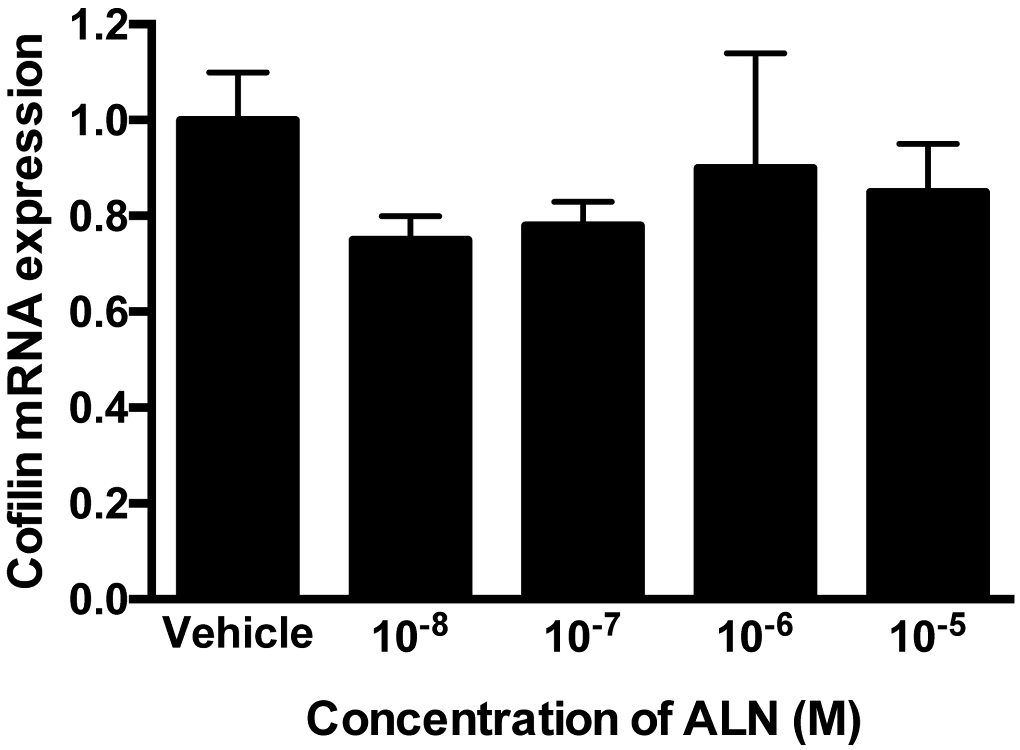

Supplementary Figure 1: The effect ALN (10^{-8}-10^{-5} M) treatment of PC-3 cells for 24 h on cofilin mRNA levels was analyzed with qRT-PCR. ALN did not affect cofilin mRNA levels.

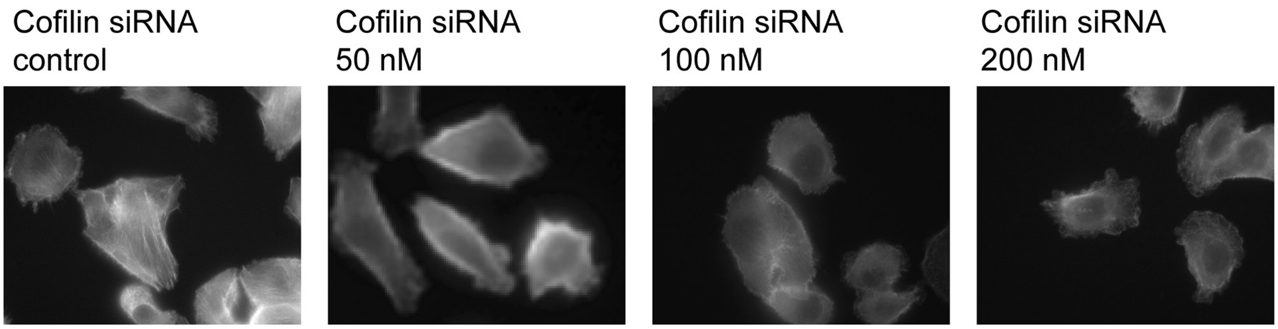

Supplementary Figure 2: Depletion of cofilin by siRNA disrupted actin stress fiber organization in PC-3 cells.
